# Improved Electrostatics through Digital Etch Schemes in Vertical GaSb Nanowire p-MOSFETs on Si

**DOI:** 10.1021/acsaelm.1c01134

**Published:** 2022-01-10

**Authors:** Zhongyunshen Zhu, Adam Jönsson, Yen-Po Liu, Johannes Svensson, Rainer Timm, Lars-Erik Wernersson

**Affiliations:** † Division of Electromagnetics and Nanoelectronics, Department of Electrical and Information Technology, Lund University, P.O. Box 117, 221 00 Lund, Sweden; ‡ Division of Synchrotron Radiation Research, Department of Physics, and NanoLund, Lund University, P.O. Box 117, 221 00 Lund, Sweden

**Keywords:** III−V, MOSFET, nanowire, GaSb, digital etch, BOE 30:1, HCl:IPA

## Abstract

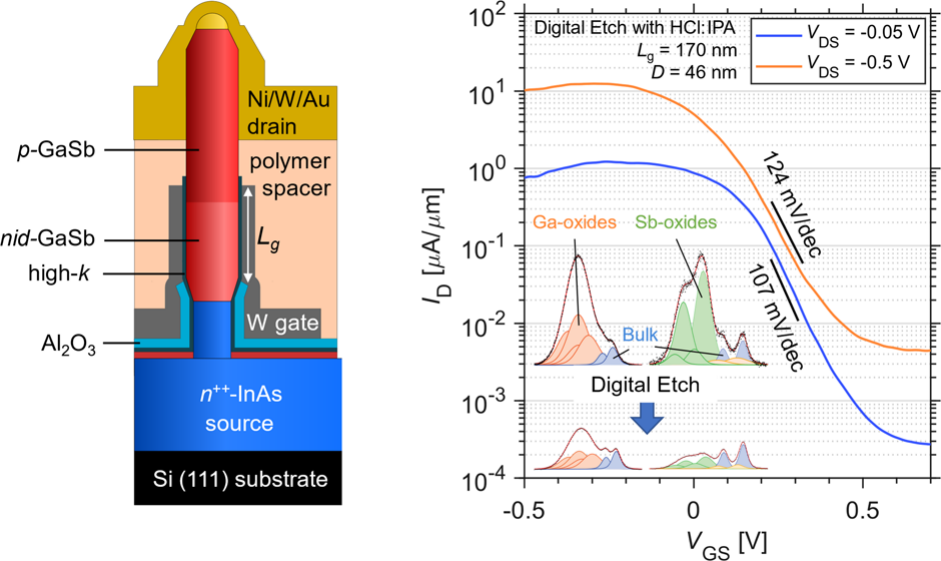

Sb-based semiconductors are critical p-channel materials for III–V complementary metal oxide semiconductor (CMOS) technology, while the performance of Sb-based metal-oxide-semiconductor field-effect transistors (MOSFETs) is typically inhibited by the low quality of the channel to gate dielectric interface, which leads to poor gate modulation. In this study, we achieve improved electrostatics of vertical GaSb nanowire p-channel MOSFETs by employing robust digital etch (DE) schemes, prior to high-κ deposition. Two different processes, based on buffer-oxide etcher (BOE) 30:1 and HCl:IPA 1:10, are compared. We demonstrate that water-based BOE 30:1, which is a common etchant in Si-based CMOS process, gives an equally controllable etching for GaSb nanowires compared to alcohol-based HCl:IPA, thereby realizing III–V on Si with the same etchant selection. Both DE chemicals show good interface quality of GaSb with a substantial reduction in Sb oxides for both etchants while the HCl:IPA resulted in a stronger reduction in the Ga oxides, as determined by X-ray photoelectron spectroscopy and in agreement with the electrical characterization. By implementing these DE schemes into vertical GaSb nanowire MOSFETs, a subthreshold swing of 107 mV/dec is obtained in the HCl:IPA pretreated sample, which is state of the art compared to reported Sb-based MOSFETs, suggesting a potential of Sb-based p-type devices for all-III–V CMOS technologies.

## 1 Introduction

Alternative channel materials supplementing Si in metal-oxide-semiconductor field-effect transistors (MOSFETs) have been studied for boosting the device performance to extend Moore’s law in semiconductor manufacturing.^1−3^ Due to the high mobility and injection velocity, III–V semiconductors such as InGaAs are promising as the channel material integrated on Si substrates.^4^ Recently, InAs/InGaAs nanowire n-type MOSFET on Si has shown great performance with a peak transconductance over 3 mS/μm.^5^ In the case of III–V p-type channel, Sb-based materials such as GaSb and InGaSb have exhibited attractive characteristics for p-MOSFETs due to their high bulk hole mobility.^6−8^ However, the main challenge of a Sb-based MOSFET to completely benefit from the high mobility of the bulk material is the poor electrostatics originating from the high level of interface and border traps. To substantially improve the electrostatic control for scaled transistors, various nanowire-based multigate architectures such as FinFETs^9^ and vertical gate-all-around (GAA) MOSFETs^10,11^ are being pursued. A key step to achieve scaled nanowire diameters or fin widths for III–V semiconductors has been to employ digital etch (DE) methods to both reduce dimensions and provide native oxide removal.^12,13^ In particular, Sb-based structures are known for rapid reoxidation leading to a high density of interface traps,^14,15^ thus strongly limiting the electrostatic control and the off-state performance for Sb-based p-MOSFETs.^16,17^ Different steps aimed toward improving the GaSb MOS interface quality have been reported, such as introducing thin InAs^18^ or InGaAs^19^ interfacial layer, hydrogen plasma pretreatment,^20^ and *in situ* gate oxide deposition, which resulted in a density of interface defects (*D*_it_) down to ∼10^12^ cm^–2^ eV^–1^.^21^ Moreover, various chemical pretreatments, including (NH_4)2_S,^22,23^ HCl:H_2_O,^14,24^ and HF:H_2_O^10^ for GaSb surface passivation have been reported. Recently, alcohol-based HCl combined with oxygen has been selected as the most stable DE scheme for heterostructural Sb-based FinFETs among different combinations of oxidations and chemicals.^25^ However, the electrostatics of the corresponding devices still need to be further improved.

In this paper, we achieve the lowest reported subthreshold swing (SS) of 107 mV/dec for GaSb-based vertical GAA nanowire MOSFETs by digital etching in HCl:IPA 1:10 (the concentration is the same for the entire paper if not specified, thus simplified in HCl:IPA in the following text and figures). In addition, we for the first time introduce buffer-oxide etcher (BOE) 30:1 into the DE process for GaSb device fabrication and find that both water-based BOE and alcohol-based HCl have controllable DE rates within five cycles for diameter reduction in GaSb nanowires. As an alternative, BOE is commonly used in the current Si-based complementary metal oxide semiconductor (CMOS) technology, allowing us to directly utilize the same chemical in Sb-based process without introducing new etchants. Furthermore, the surface quality of GaSb pretreated by BOE 30:1 and HCl:IPA is evaluated by X-ray photoelectron spectroscopy (XPS), which exhibits a remarkable reduction in Sb oxides for both cases while more Ga oxides are removed in the HCl:IPA sample. This result agrees well with the electrical characterization that SS is relatively lower in the MOSFETs pretreated in HCl:IPA. In both cases, a noticeably reduced SS in GaSb nanowire MOSFETs in this work compared to previous reports regarding Sb-based transistors indicates a better electrostatic control.

## 2 Results and Discussion

### 2.1 MOSFET Structure and Performance

**Figure 1:**
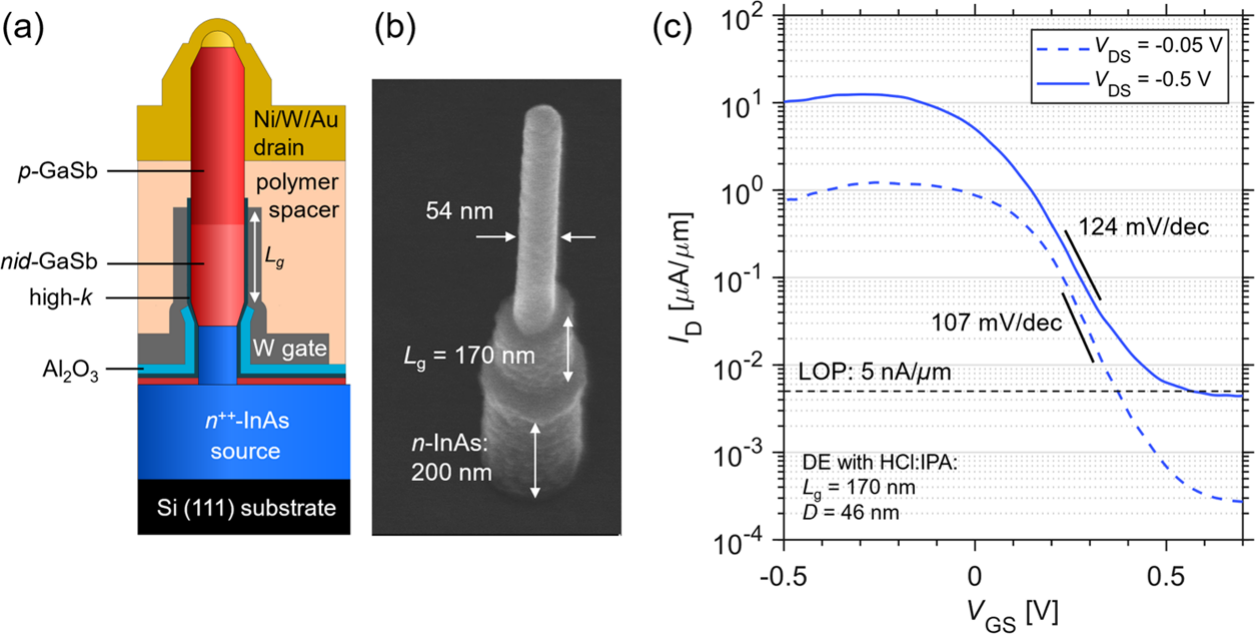
Electrical characterization of the device with lowest SS. (a) Schematic of a single GaSb nanowire MOSFET with digital etch as the first step of the fabrication. (b) Scanning electron microscopy (SEM) image of a single nanowire device after gate length definition. The measured diameter includes the nanowire and 4 nm gate oxides. (c) Transfer characteristics of the nanowire device with two-cycle DE using HCl:IPA 1:10 just before high-κ deposition.

Figure 1a illustrates the schematic of a single nanowire MOSFET with the GAA architecture. The growth of InAs-GaSb nanowire growth was done by metal–organic vapor-phase epitaxy (MOVPE) via the vapor–liquid–solid (VLS) process (see the Experimental Methods section for details). The device fabrication started from the bottom with DE using BOE 30:1 or HCl:IPA and high-κ deposition [equivalent oxide thickness (EOT) is ∼1 nm, and the relative permittivity is ∼22] as well as the bottom spacer Al_2_O_3_, which was partially removed on the nanowire top (see Figure 1a). The gate metal was then deposited, and the gate length (*L*_g_) was defined by the vertical photoresist mask, which was back-etched by oxygen plasma, followed by the gate metal etch on the nanowire sidewalls shown in Figure 1b. The nanowire diameter in the channel region is 46 nm. By optimizing the DE condition of GaSb nanowire MOSFETs, a device with DE using HCl:IPA exhibits the minimum SS (SS_min_, minimum point SS) down to 107 mV/dec at *V*_DS_ = −0.05 V, as shown in Figure 1c, in addition to a minimum off-current (*I*_off_) at *V*_DS_ = −0.5 V approaching 4 nA/μm, which meets the *I*_off_ specification under low operation power (LOP) condition defined by International Technology Roadmap for Semiconductors (ITRS)^26^ for low power logic applications.

### 2.2 Digital Etch Comparison

**Figure 2:**
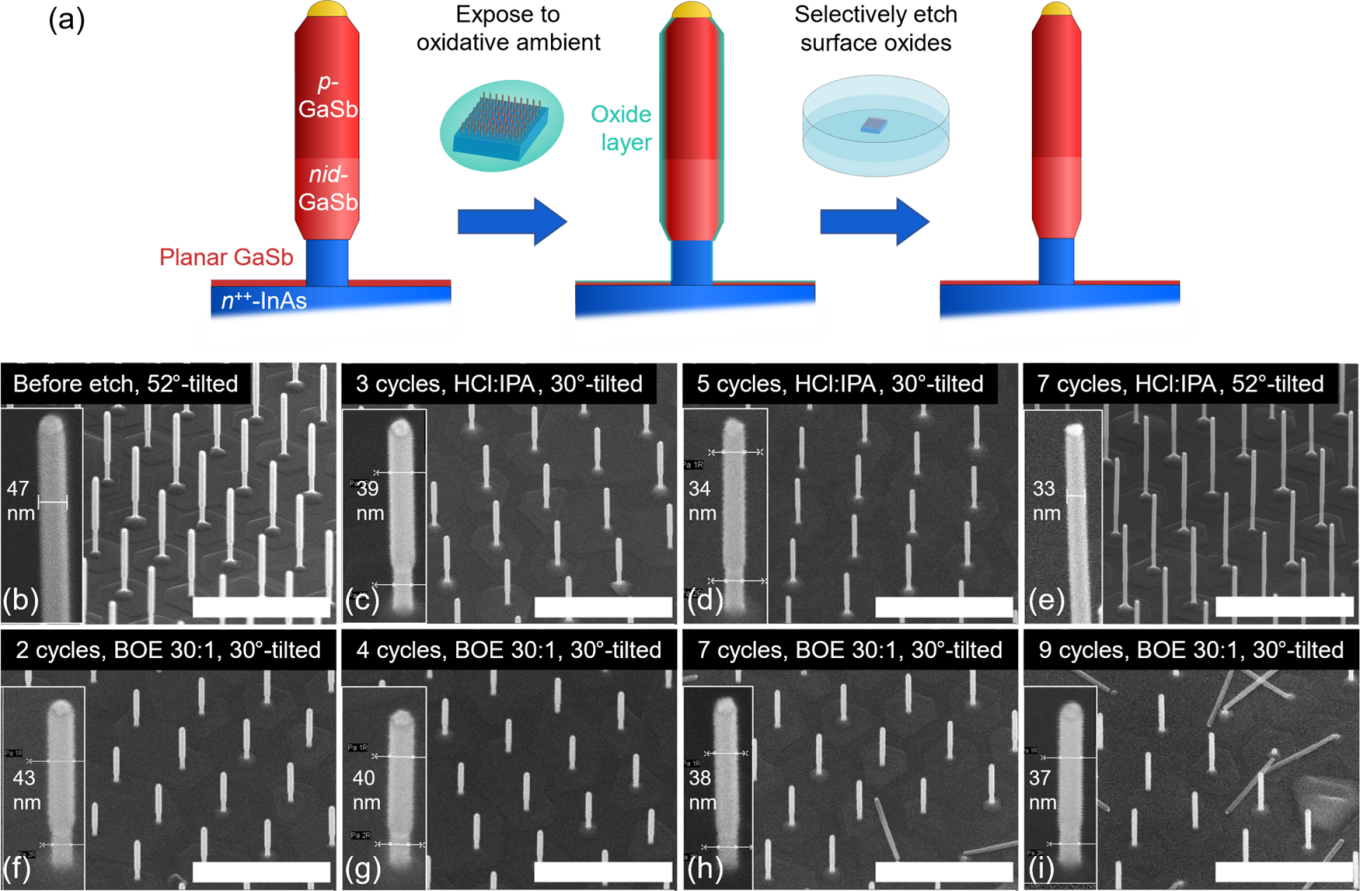
(a) Schematics of the one-cycle digital etch process. Evolution of a GaSb nanowire array in a sequential etch experiment with different numbers of DE cycles in (c–e) HCl:IPA and (f–i) BOE 30:1, respectively, performed in different samples. (b) SEM image of nanowires before DE. The insets show a single nanowire in the array. The scale bars are 1 μm.

**Figure 3:**
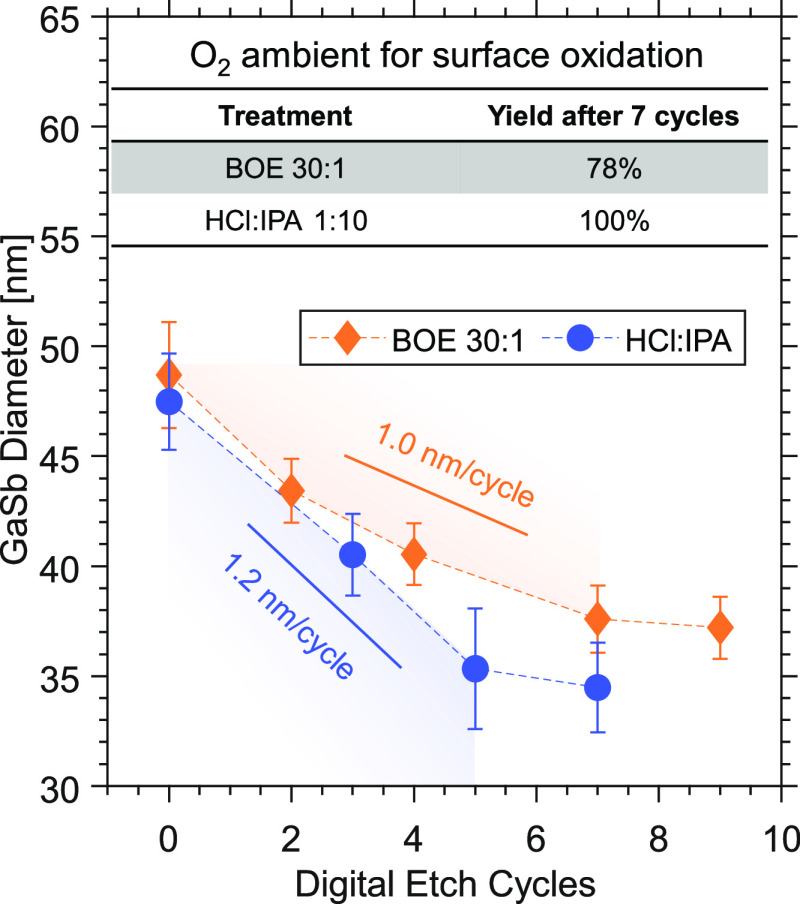
Comparison of GaSb nanowire diameter with the number of DE cycles in BOE 30:1 and HCl:IPA. The inset table shows the yield after seven cycles comparing DE with BOE 30:1 or HCl:IPA.

Figure 2a shows the schematics of the DE process where the nanowires are first oxidized in a O_2_ chamber and then wet-etched by either BOE 30:1 or HCl:IPA (see details in the Experimental Methods section). Since oxidation occurs easily for a GaSb surface,^15^ the exposure of conventional dry oxidations such as ozone^27^ and oxygen plasma^12^ would produce a great amount of higher-order Sb oxides such as Sb_2_O_5_, which are difficult to completely etch in most acids.^25^ When a critical amount of Sb_2_O_5_ has formed at the surface, etching is inhibited. Therefore, O_2_ is selected as a gentle oxidizer for GaSb to mitigate the presence of Sb_2_O_5_. The oxidation time was selected as 8 min based on our optimizing experiments (see Supporting Information Figure S4). The wet etch process selectively removes the surface oxides on the nanowire sidewalls, thereby reducing the diameter of the nanowires. The etch stops after about 30 s when no more etch was observed when increasing the etch time (see Supporting Information Figure S4). Figure 2b–i demonstrates the thinning process of nanowires with an original diameter of 48 ± 3 nm after different numbers of DE cycles comparing two different samples with DE using BOE 30:1 or HCl:IPA. In both cases, the nanowire diameter is gradually reduced by repeated DE steps while the etch is inhibited after five cycles in HCl:IPA and seven cycles in BOE 30:1 (see both Figures 2 and 3). Notably, due to strong forces caused by surface tension the nanowires break as they become thin. The nanowires break after 7 cycles when using BOE 30:1, but in contrast, the nanowires are stable after the same number of cycles using HCl:IPA. The reason that a lower yield is found in the case of BOE 30:1 (see inset table of Figure 3) can be water-based acids have higher surface tension compared to alcohol-based acids,^28^ which causes nanowires breaking off either during the wet etch step or the following rinsing step.^13^

Figure 3 illustrates the diameter evolution with consecutive DE steps using BOE 30:1 and HCl:IPA based on the nanowire array shown in Figure 2b–i. The etch rate is 1.2 nm/cycle for the first two cycles for both etchants. However, after two cycles, the etch rate is gradually reduced in the case of BOE 30:1 but still retains when etching in HCl:IPA until five cycles. The average etch rate in BOE 30:1 is ∼1 nm/cycle until the seventh cycle. The potential reason for the etch rate drop earlier in BOE 30:1 is that reoxidation occurs more likely in water-based BOE compared to alcohol-based HCl. However, in both cases, after many cycles of the DE (7 cycles of BOE 30:1 and 5 cycles in HCl:IPA), the etch rate decreases dramatically perhaps attributed to the emergence of insoluble oxides on the nanowire surface, which restrains the DE process. This implies that too many cycles of the DE can probably degrade the channel surface. Thus, a combination of thinner nanowires from growth and one cycle or two cycles of DE could provide better electrostatics benefiting from both lower *D*_it_ and geometry.

### 2.3 Electrical Characterizations of GaSb Nanowire MOSFETs

**Figure 4:**
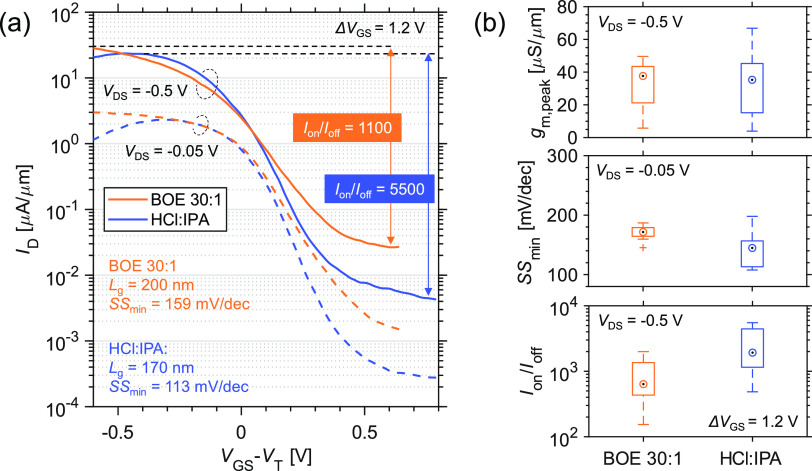
Electrical data of individual GaSb nanowire p-channel MOSFET with different surface pretreatment. (a) Transfer characteristics of the devices with two different DE processes for the channel. *I*_on_ and *I*_off_ are taken from the maximum and minimum *I*_D_ in the given gate bias range Δ*V*_GS_ = 1.2 V, respectively. Here, an overdrive voltage *V*_GS_ – *V*_T_ was selected to compare the transfer characteristics in two cases. (b) Statistical result with boxplots including *g*_m,peak_, SS_min_, and *I*_on_/*I*_off_ based on 10 devices. SS_min_ is taken from the minimum point SS at *V*_DS_ = −0.05 V.

Figure 4a compares the transfer characteristics of two samples digitally etched by BOE 30:1 and HCl:IPA, respectively, for two cycles. We believe that the difference in *L*_g_ between two samples (200 vs 170 nm) has a negligible impact on the characteristics since one can consider both samples as long-channel devices regarding the corresponding channel diameter, which is 46 nm for the sample digitally etched in HCl:IPA and 47 nm for the sample digitally etched in BOE 30:1 (see SEM result in Supporting Information Figure S2). The channel diameter is verified to be identical to that of the top GaSb nanowire segment (see Figure 2 and Supporting Information Figure S2). Therefore, the performances in two samples can be compared and the impact of nanowire diameter can be excluded. Thus, the main effect is believed to originate from the interface properties. Although almost identical on-current (*I*_on_) in both individual devices with different channel pretreatments is found at *V*_DS_ = −0.5 V, the *I*_off_ in HCl:IPA pretreated sample is about 5 times lower than that in BOE 30:1 pretreated sample. Thus, *I*_on_/*I*_off_ = 5500 (see the definition of *I*_on_ and *I*_off_ for on–off current ratio in Figure 4 caption) obtained in the sample with DE in HCl:IPA is also 5 times higher than that in the case of BOE 30:1. Furthermore, a lower SS_min_ of 113 mV/dec is achieved in the sample with DE in HCl:IPA than that of BOE 30:1 pretreated sample with SS_min_ = 160 mV/dec. It is found that *I*_D_ drops at high gate bias in the device with DE in HCl:IPA probably due to the presence of source depletion attributed to that the gate overlaps the 200 nm long InAs segment. However, no source depletion is observed in the device with DE in BOE 30:1 since the InAs segment is shorter (see SEM images and output characteristics of two samples in Supporting Information Figure S2). It should be noticed that in Figure S2d, the output characteristics of HCl:IPA pretreated device show a series resistance existing at a high gate bias and a low source–drain bias mainly due to the source depletion effect. Thus, the on-resistance of the device with DE in HCl:IPA is higher than that in the sample with DE in BOE 30:1.

Figure 4b shows multiple statistics including peak transconductance (*g*_m,peak_), SS_min_, and *I*_on_/*I*_off_ for two samples based on about 10 devices. Similar to the individual device comparison in either case, the on-state performance is similar in two samples, whereas improved off-state performance exists in the sample pretreated in HCl:IPA, resulting in a higher *I*_on_/*I*_off_ and smaller SS_min_ on average, which can be mainly attributed to lower *D*_it_ between the channel and high-κ. *D*_it_ affects the subthreshold swing as follows:^29^ SS ≈ (*kT*/*q*)·ln(10)(1 + *qD*_it_/*C*_ox_), where *k* is the Boltzmann constant, *T* the temperature, *q* the electron charge, and *C*_ox_ the oxide capacitance. Since both samples have identical gate stacks and similar channel diameters (approximately the same *C*_ox_), it is possible to estimate the *D*_it_ difference by comparing the statistical result of SS_min_. In contrast to the sample digitally etched in BOE 30:1, the HCl:IPA pretreated sample has an approximately 18% lower median value of SS_min_ at *V*_DS_ = −0.05 V, corresponding to a 27% lower *D*_it_. In the case of the individual device in two samples with similar on-performance as shown in Figure 4a, the difference in *D*_it_ between two devices, however, reaches ∼46%. The degraded MOS interface in the sample with DE in BOE 30:1 mainly originates from excessive Ga oxides at the GaSb channel surface, which we will discuss in the next section. Nevertheless, the variation in performance among devices is lower in the case of BOE 30:1 than that with the DE in HCl:IPA, probably partially attributed to higher uniformity of nanowire diameter after the DE in BOE 30:1 (only ±1 nm after 4 cycles, compared to ±2 nm after 3 cycles for HCl:IPA) even for two cycles (which we used for real nanowire transistors) as shown in Figure 3.

### 2.4 Surface Composition of GaSb with Different Pretreatments

**Figure 5:**
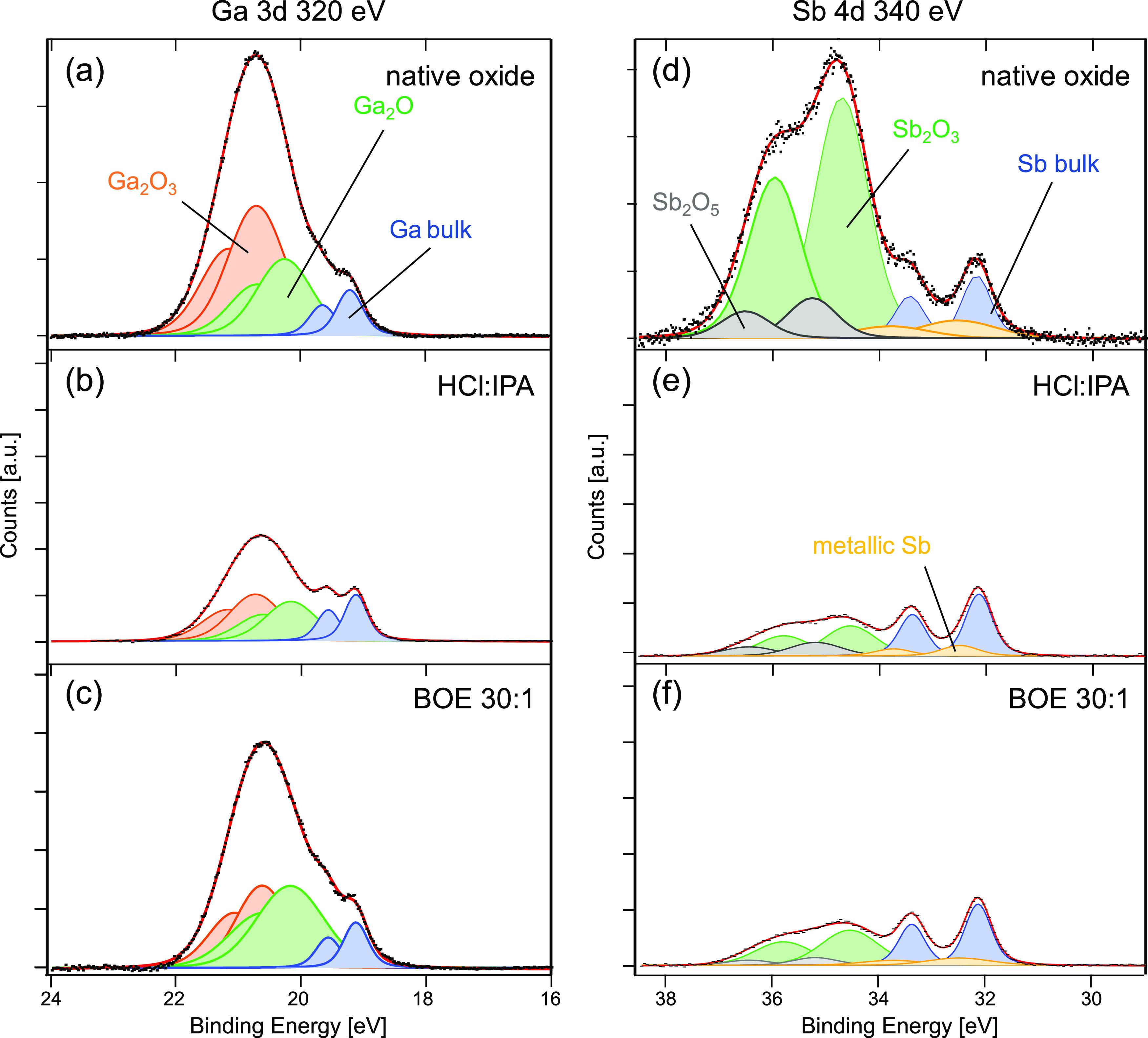
XPS data of (a–c) Ga 3d and (d–f) Sb 4d core levels, obtained from (a, d) the GaSb(100) substrate with native oxide (no surface pretreatment); (b, e) a GaSb(100) substrate after HCl:IPA 1:10 etching followed by ALD; and (c, f) a GaSb(100) substrate after BOE 30:1 etching followed by ALD. Raw data are displayed as black dots and fitted spectra as red lines, individual fitted doublet components are indicated. To visualize the oxide removal, intensities from all samples are normalized to the peak heights of the Ga bulk and Sb bulk components, respectively.

To further explore the origin of the differences in electrical performance and relate them to differences regarding the material properties of the GaSb/high-κ interface, synchrotron-based XPS was performed on GaSb(100) substrates with either BOE 30:1 or HCl:IPA DE followed by atomic layer deposition (ALD), using the same etching and ALD process as for the nanowire device samples, and on another GaSb(100) substrate with native oxide as a reference. It should be noted that the as-grown GaSb nanowires are terminated by (110) side facets. However, after the digital etching, we expect a rather rounded shape of the nanowires, consisting of many small terraces of different surface orientations. Previous studies on InAs nanowires have shown that their oxide composition was comparable with that of InAs(100) substrates. On the other hand, XPS studies on nanowire samples suffer from very low count rates. Therefore, we chose GaSb(100) substrates for the quantitative surface characterization in this work.^32^ Ga 3d and Sb 4d core-level spectra are shown in Figure 5. Three doublets are needed for consistently fitting the Ga 3d spectra (see Figure 5a–c), with a bulk component corresponding to Ga–Sb bonds at a binding energy of 19.1 eV (blue) and additional doublets at a binding energy shift of +1.05 eV (green) and +1.5 eV (orange), respectively. The latter components can be related to Ga^1+^ as in Ga_2_O and to Ga^3+^ as in Ga_2_O_3_, in agreement with the literature.^20,30^ The spectrum of the reference sample with native oxide, Figure 5a, is dominated by the Ga^3+^ peak, and also a significant amount of Ga^1+^ can be seen, in addition to Ga bonding to Sb (Ga bulk peak). The Ga-oxide components are not removed upon DE and ALD, confirming the rather weak ALD self-cleaning effect for GaSb^20,31^ compared to, e.g., InAs,^32,33^ but significant differences can be seen after the two different DE processes, as the HCl:IPA sample contains much less Ga oxides than the BOE 30:1 sample.

For the Sb 4d spectra, shown in the right column of Figure 5, four doublets are needed for a consistent fit: the bulk component, corresponding to Sb–Ga bonds, is observed at a binding energy of 32.1 eV (blue), and additional doublets are found with binding energy shifts of +0.36 eV (yellow), +2.42 eV (green), and +3.07 eV (gray), respectively. The component at + 0.36 eV is assumed to be Sb in a 0 oxidation state, as is the case for metallic Sb, and also for atomic-scale defects such as Sb antisites. The other two components are due to Sb oxides, in accordance with the literature,^30,34,35^ with Sb^3+^ as in Sb_2_O_3_ at +2.42 eV (green). The higher oxidation state at +3.07 eV has by most authors been considered as Sb^5+^ corresponding to Sb_2_O_5_,^30,34^ while McDonnell et al. have argued that a Sb^4+^ state with Sb_2_O_4_ should be thermodynamically favorable over Sb_2_O_5_ in a GaSb-oxide environment.^35^ Here, we label the component as Sb^5+^ or Sb_2_O_5_ but want to point out that we cannot exclude that it is Sb^4+^ and Sb_2_O_4_ instead, which, however, would not change our conclusions. The spectra show that the amounts of both Sb oxides and metallic Sb are significantly reduced after DE with either HCl:IPA or BOE 30:1 followed by ALD. The comparison of the two different DE processes indicates that the HCl:IPA sample contains less Sb_2_O_3_ but slightly more Sb_2_O_5_.

A reduction in the amount of Sb oxides combined with an increase of Ga oxides has been reported before for high-κ ALD on GaSb^31,36^ and also for annealing oxidized GaSb at moderate temperatures^35^ since the Ga oxides are energetically more stable than the Sb oxides.^20,31,35^ Here, we see a strong reduction of Sb oxides upon DE and ALD, both for the HCl:IPA and BOE 30:1 samples and also a significant reduction in the amount of Ga oxides for the HCl:IPA sample and at least a moderate reduction in the amount of Ga oxides for the BOE 30:1 sample. The strongly reduced amount of Sb oxides can be considered as the main reason for the highly improved electrostatics (better SS) of the GaSb nanowire devices, as it turns the GaSb/high-κ interface from being Sb-rich, which is detrimental for device performance,^36^ to Ga-rich. It is noteworthy that a significant amount of Sb^0^ is observed in all samples, while dramatically reduced after DE and ALD. In the GaAs and InAs material systems, the As^0^ state stands for As–As bonds, which are considered as the interface defect with the worst impact on devices, especially in GaAs where these states are situated within the band gap.^37^ However, Sb–Sb bonds, which are indicated by Sb^0^ states, are located within the GaSb conduction band,^37^ thereby being less relevant for p-type GaSb devices operated in the hole conductance regime.

Let us now focus on the differences in the interface composition between the HCl:IPA and the BOE 30:1 sample. We quantify the amount of interface oxide by obtaining the area under the doublet peak of a fitted component and dividing it by the area of the bulk peak. The absolute values of these oxide component ratios, which are shown in Table 1, are dependent on many parameters including surface chemical composition and material properties and also photon energy and properties of the beamline optics, but a relative comparison of the ratios is meaningful. Accordingly, we can conclude that the BOE 30:1 sample contains more of both types of Ga oxides and Sb_2_O_3_ as the HCl:IPA sample. This is likely the main reason for the higher *D*_it_ of the BOE 30:1 sample, which agrees well with our electrical result that shows lower SS in the devices with DE in HCl:IPA. The great amount of Ga oxides in BOE 30:1 likely originates from the insufficient removal during the DE or reoxidation in water-based BOE^14^ either during the etching step or the following rinse (in deionized water) step. The slightly larger amount of high Sb-oxide states (Sb_2_O_5_) existing in the sample pretreated by HCl:IPA seems to be less relevant.

**Table 1:** Relative Intensity Ratios of Different Ga 3d and Sb 4d Components Normalized by the Corresponding Bulk (GaSb) XPS Signal Intensities^a^

Ga 3d	Ga_2_O/bulk	Ga_2_O_3_/bulk
native oxide	3.24	5.64
Ga HCl:IPA	1.39	2.43
Ga BOE 30:1	4.43	3.50

Sb 4d	metallic/bulk	Sb_2_O_3_/bulk	Sb_2_O_5_/bulk
native oxide	0.66	6.66	1.10
Sb HCl:IPA	0.22	0.85	0.38
Sb BOE 30:1	0.26	1.14	0.20

a The intensity of a doublet component is determined by the peak height and width, as fitted from the corresponding core-level spectrum.

### 2.5 Discussion and Benchmarking

**Figure 6:**
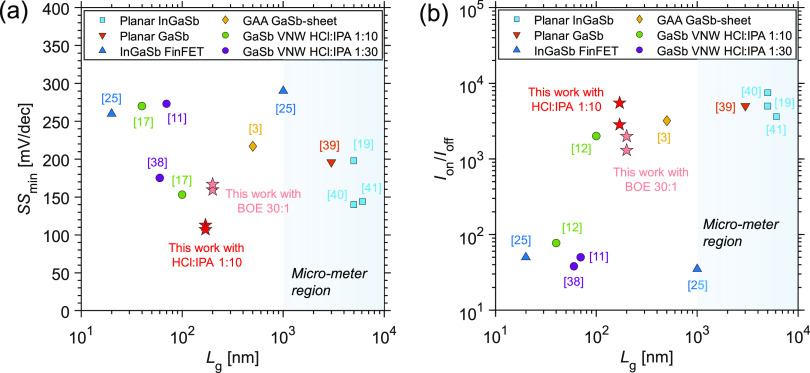
Benchmarking of our devices against other III-Sb devices in (a) SS_min_ vs *L*_g_ and (b) *I*_on_/*I*_off_ vs *L*_g_. The blue region indicates the devices with *L*_g_ > 1 μm. Note that Δ*V*_GS_ differs in *I*_on_/*I*_off_ comparison. Refs (3, 11, 17, 19, 25, 38−41).

Finally, benchmarking to the state-of-the-art Sb-based p-MOSFETs with various device structures and channel lengths is presented in Figure 6. Our vertical nanowire devices with DE using either BOE 30:1 or HCl:IPA exhibit a competitive performance in SS and *I*_on_/*I*_off_, in Figure 6a,b, respectively. It is notable that SS in our devices is lower compared to both longer- and shorter-channel devices, while *I*_on_/*I*_off_ reaches a similar value as long-channel devices, which indicates a good off-current. One of the reasons is that our vertical nanowire devices benefit from the GAA architecture that provides great electrostatics for gate modulation. However, compared to other GAA GaSb MOSFETs or our previous vertical GAA transistors fabricated with different DE techniques and process flows (see Figure 6), devices in this work still perform better. This suggests that the surface pretreatment in this work also plays an important role to further improve the electrostatics of the MOSFETs.

## 3 Conclusions

We have improved the electrostatics of GaSb p-type MOSFETs by pretreating the channel surface through the DE with HCl:IPA or BOE 30:1 prior to the high-κ deposition, achieving the lowest SS_min_ down to 107 mV/dec as well as an increased *I*_on_/*I*_off_ over 3 orders of magnitude. The DE comparison of GaSb nanowires shows that HCl:IPA provides a slightly higher etch rate while the DE in both cases stops after a specific number of cycles probably due to the existence of insoluble oxides. This implies that the *D*_it_ may increase again at the surface when applying too many cycles of the DE. The electrical characterizations in addition to XPS results consistently show that alcohol-based HCl:IPA pretreated sample has a relatively lower *D*_it_ compared to water-based BOE 30:1. Despite slightly lower off-state performance in the MOSFETs, BOE 30:1 can still be considered as an alternative for the surface passivation of Sb-based devices, particularly when involved in Si-based CMOS processing. But in general, the DE using HCl:IPA gives further improved electrostatics in Sb-based devices for all-III–V CMOS technology.

## 4 Experimental Methods

### 4.1 Nanowire Epitaxy

Heterostructure InAs-GaSb nanowires are grown on Si substrates with a 260 nm thick n^++^-InAs buffer layer, from prepatterned Au gold dots, by MOVPE via the VLS process. The nanowire growth begins by employing a short Sn-doped InAs system with precursors of trimethylindium (TMIn) and arsine (AsH_3_) (molar fraction: χ_TMIn_ = 6.1 × 10^–6^, TESn/TMIn = 4) to provide better nucleation for the subsequent GaSb nanowire growth where trimethylgallium (TMGa) and trimethylantimony (TMSb) are used as precursors. The nonintentionally doped (nid) GaSb with background doping of ∼10^16^ cm^–3^ and Zn-doped p-type GaSb (molar fraction: χ_TMGa_ = 4.9 × 10^–5^, DEZn/TMGa = 0.39) are subsequentially grown at 515 °C, providing the channel and drain material, respectively.

### 4.2 Device Fabrication

The device fabrication is initialized, directly after growth, by digital etching using oxidation in O_2_ ambient for 8 min followed by dipping the sample in either HCl:IPA 1:10 or BOE 30:1 for 30 s. Then, the samples were rinsed for 60 s in IPA for HCl:IPA etch and DI water for BOE 30:1 etch and dried by flowing N_2_. Then, the above steps were repeated for another DE cycle. Directly after the surface treatment (within seconds), atomic layer deposition (ALD) is performed consisting of a bilayer high κ with Al_2_O_3_/HfO_2_ (1/3 nm, EOT ≈ 1 nm, negligible gate leakage) with an added 20 nm thick Al_2_O_3_ film as the bottom (first) spacer. A flat sidewall surface was observed after the ALD-deposited high-κ and spacer Al_2_O_3_, indicating a smooth interface quality of dielectric layer (see Supporting Information Figure S3a). The bottom spacer is finalized by selectively etching the top segment of the 20 nm thick Al_2_O_3_ using a back-etched S18 mask and HF 1:400 etch. The gate is then defined using a 60 nm sputtered W aligned via a similar S18 back-etch mask now followed by dry etching (SF6:Ar) which sets the final gate length. Both the nanowire diameter of the channel and the gate length are verified by scanning electron microscopy (SEM) imaging (see Supporting Information Figure S2). The samples are finalized by second spacer deposition and contact metallization (Ni/W/Au).

### 4.3 XPS Characterization

Surface chemistry was characterized by synchrotron-based XPS at the FlexPES beamline of the MAX IV Laboratory, Sweden, as well as at the SuperESCA beamline of the ELETTRA synchrotron, Italy. All of the characterizations were performed on commercial GaSb(100) wafers. Bulk substrates were prioritized over nanowire samples for the XPS measurements due to the much higher signal intensity and better signal-to-noise ratio.^32^ The reference sample has native oxides on the surface without any pretreatment while either BOE 30:1 or HCl:IPA pretreated sample was followed by ALD, using the same recipe as for the nanowire devices. Ga 3d and Sb 4d core levels were obtained at photon energies of 320 and 340 eV, respectively, resulting in the same kinetic energy of 300 eV, corresponding to an inelastic mean free path of 0.85 nm. XPS data were fitted using the Igor Pro software, assuming Voigt peak shapes and a polynomial background. A Lorentzian width of 0.18 eV (0.22 eV), a spin–orbit splitting of 0.44 eV (1.25 eV), a doublet ratio of 0.67 (0.67), and Gaussian widths within 0.3–1.2 eV (0.5–1.3 eV) were obtained for the Ga 3d (Sb 4d) peaks.

## Supporting Information Available

Direct etch methods of GaSb nanowires, more scanning electron microscopy images of nanowires, output characterizations of devices in two samples, and the optimizing experiments of digital etch conditions (PDF)
