# Supplementary material for: Improved Electrostatics through Digital Etch Schemes in Vertical GaSb Nanowire p-MOSFETs on Si
Source: ACS Appl Electron Mater. 2022 Jan 10;4(1):531–8. doi: 10.1021/acsaelm.1c01134 (PMC8793030; doi:10.1021/acsaelm.1c01134)
Supplement: Supplementary file 1 — el1c01134_si_001.pdf [file el1c01134_si_001.pdf]

Supporting Information

Improved Electrostatics through Digital Etch Schemes in Vertical GaSb Nanowire p-MOSFETs on Si

Zhongyunshen Zhu[>], Adam Jönsson[>], Yen-Po Liu[>>], Johannes Svensson[>], Rainer Timm[>>], and Lars-Erik Wernersson[>]*

[>] *Division of Electromagnetics and Nanoelectronics, Department of Electrical and Information Technology, Lund University, BOX 117, 221 00, Lund, Sweden*

[>>] *Division of Synchrotron Radiation Research, Department of Physics, and NanoLund, Lund University, BOX 117, 221 00, Lund, Sweden*

* Corresponding Author: zhongyunshen.zhu@eit.lth.se

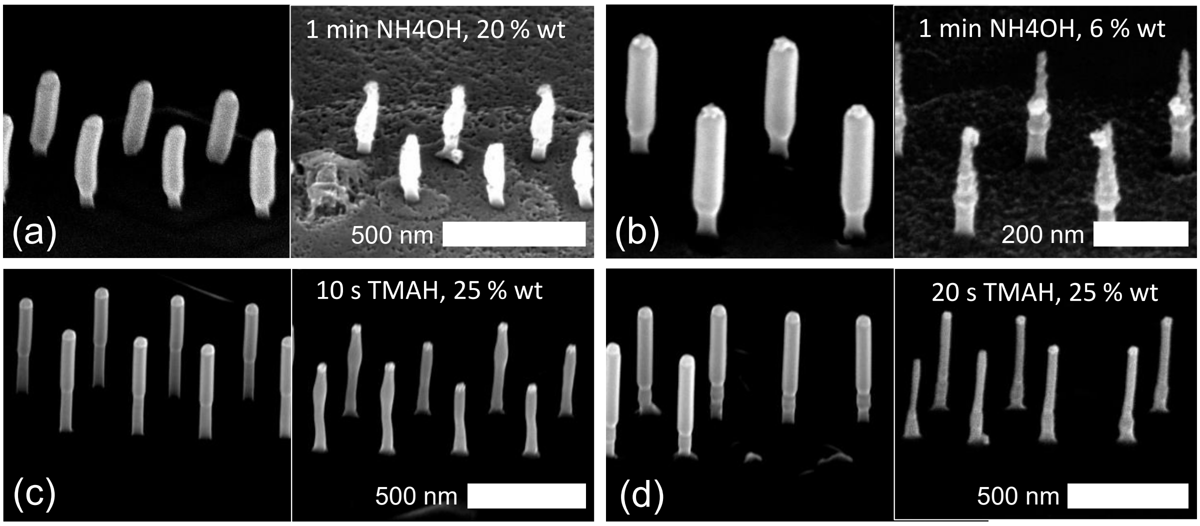

Figure S1. Various direct wet etch process for GaSb selectively to InAs with different chemicals including NH_4OH [(a) and (b) with different concentrations] and 25% wt. TMAH

(Tetramethylammonium hydroxide) [(c) and (d) with different etch time]. However, they are either too aggressive or uncontrollable for nanoscale GaSb vertical NWs. Therefore, a way to precisely etch the NW to a certain diameter is required. Digital etch process can be one of good options.

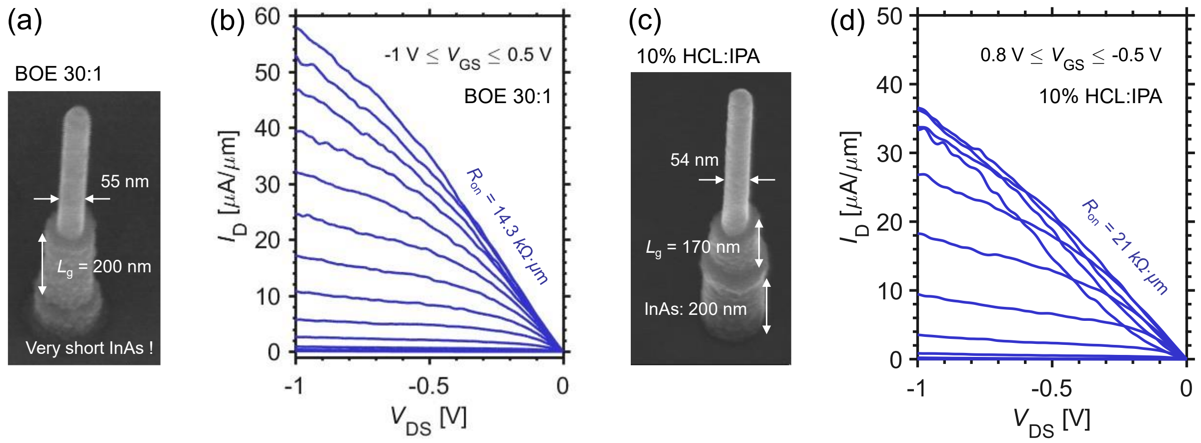

Figure S2. SEM image of the single NW device after gate length definition in 2 samples with different digital etch scheme: (a) BOE 30:1, and (c) HCl:IPA 1:10. Note that measured diameters in SEM include 4 nm high-κ layer, which corresponds to 47 nm and 46 nm in (a) and (c), respectively. Their corresponding output characteristics are shown in (b) and (d), respectively. Long InAs segment in device pretreated by HCl:IPA leads to the source depletion in both transfer (see main text) and output characteristics which gives greater on-resistance (R_{on}).

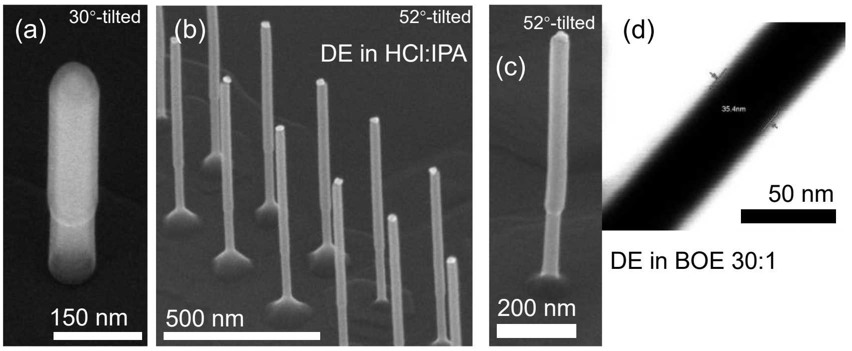

Figure S3. (a) SEM image of nanowires after high-*k* (1-nm Al_2O_3 and 3-nm HfO_2) and first spacer (20-nm Al_2O_3) deposition, showing a flat surface and good quality of as-deposited dielectric layer. (b) and (c) are SEM images of DE in HCl:IPA 1:10 and BOE 30:1, respectively. (d) Scanning transmission electron microscopy (STEM) image of a nanowire with DE in BOE 30:1. Both SEM and STEM show a flat surface in the sample pretreated in either HCl:IPA or BOE 30:1, as well as a uniform sidewall surface of the entire GaSb nanowire.

	8 min in O2	16 min in O2
30 s in BOE 30:1	~1.0 nm/cyc	NA
40 s in BOE 30:1	~1.0 nm/cyc	NA
30 s in HCl:IPA 1:10	~1.2 nm/cyc	~1.6 nm/cyc

Figure S4. Optimizing experiments on different DE conditions. Etch time longer than 30 s does not show more etch on the nanowire, indicating DE mechanism. Longer oxidation time will slightly increase the etch rate per cycle. However, to more accurately control the DE on the nanowire, we conclude that the optimal DE condition would be 8 min exposure in O_2 ambient and DE in the corresponding chemical for 30 s.
